# ﻿*Cryptotermes mobydicki* (Isoptera, Kalotermitidae), an extraordinary new termite species from French Guiana

**DOI:** 10.3897/zookeys.1258.166021

**Published:** 2025-11-06

**Authors:** Rudolf H. Scheffrahn, Aleš Buček, David Sillam-Dussès, Jan Šobotník

**Affiliations:** 1 Fort Lauderdale Research and Education Center, University of Florida, 3205 College Avenue, Davie, FL 33314, USA University of Florida Davie United States of America; 2 Biology Centre of the Czech Academy of Sciences, Institute of Entomology, České Budějovice, Czech Republic Biology Centre of the Czech Academy of Sciences, Institute of Entomology České Budějovice Czech Republic; 3 University Sorbonne Paris Nord, Laboratory of Experimental and Comparative Ethology, LEEC, UR 4443, F-93430, Villetaneuse, France University Sorbonne Paris Nord Villetaneuse France; 4 Faculty of Tropical AgriSciences, Czech University of Life Sciences, Kamýcká 129, 165 21 Prague 6–Suchdol, Czech Republic Czech University of Life Sciences Prague Czech Republic

**Keywords:** Amazonia, frontal process, soldier

## Abstract

*Cryptotermes mobydicki***sp. nov.** is described from the soldier caste. The soldier is unique among all *Cryptotermes* species worldwide for its very narrow, elongate head capsule terminating with an extended frontal process. The new species constitutes the sixteenth *Cryptotermes* species for South America. Our phylogenetic reconstruction indicates that *C. mobydicki***sp. nov.** is most closely related to the Neotropical and Central American *C. mangoldi*, *C. parvifrons*, *C. cymatofrons*, *C. rotundiceps*, and *C. cavifrons*.

## ﻿Introduction

The cosmopolitan genus *Cryptotermes* Banks, 1906, with 73 species (Constantino 2020a; Scheffrahn and Vasconcellos 2023), is the third most specious kalotermitid genus worldwide behind *Glyptotermes* Froggatt, 1987 and *Neotermes* Holmgren, 1911 (Krishna et al. 2013). An exceptional diversity of *Cryptotermes*, with 26 species occurs in the Caribbean Basin (Bacchus 1987; Scheffrahn 1993, 2018; Scheffrahn et al. 1998, 1999; Scheffrahn et al. 2006). *Cryptotermes* contains many economically important kalotermitid pests (Constantino 2002; Lee et al. 2024) including the invasive *C. brevis* (Walker, 1853), *C. domesticus* (Haviland, 1898), *C. dudleyi* Banks, 1918, and *C. havilandi* (Sjöstedt, 1900).

South America, including the continental islands of Trinidad and Tobago, is habitat to 13 endemic *Cryptotermes* species: *C. aequacornis* Scheffrahn & Křeček, 1999; *C. brevis* (Walker, 1853); *C. camelus* Scheffrahn, 2021; *C. chacoensis* Roisin, 2003; *C. colombianus*Casalla et al., 2016; *C. contognathus* Constantino, 2000b; *C. cubicoceps* (Emerson, 1925); *C. cylindroceps* Scheffrahn & Křeček, 1999; *C. fatulus* (Light, 1935) (new locality from mainland Ecuador; see Scheffrahn 2019); *C. mangoldi* Scheffrahn & Křeček, 1999; *C. pugnus* Scheffrahn & Vasconcellos, 2023; *C. rhicnocephalus* Bacchus, 1987; and *C. verruculosis* (Emerson, 1925). Additionally, two exotic species, *C. dudleyi* Banks, 1918 and *C. havilandi* (Sjöstedt, 1900), are established in the Neotropics (Krishna et al. 2013).

We herein describe *C. mobydicki* sp. nov. as the fourteenth endemic and sixteenth *Cryptotermes* species overall from South America.

## ﻿Material and methods

### ﻿Illustrations and measurements

Photomicrographs were taken as multi-layer montages using a Leica M205C stereomicroscope controlled by Leica Application Suite v. 3 software. Preserved specimens were taken from 85% ethanol and suspended in a pool of Purell® Hand Sanitizer to position the specimens on a transparent Petri dish background.

### ﻿Phylogenetic reconstruction

The phylogenetic tree of *Cryptotermes* + *Procryptotermes* in this study was extracted by pruning non-*Cryptotermes* and non-*Procryptotermes* species from a Kalotermitidae summary support time-calibrated phylogenetic tree reconstructed in Buček et al. (2022). See Buček et al. (2022) for further details of the phylogenetic reconstruction methods.

## ﻿Results

### ﻿Phylogenetic position

*Cryptotermes mobydicki* sp. nov. was previously included as an unknown *Cryptotermes* species “*Cryptotermes* sp. 2” in the molecular phylogenetic reconstruction of Kalotermitidae (Buček et al. 2022). *Cryptotermes mobydicki* sp. nov. is in the mitochondrial genome-based phylogeny related to other endemic Neotropical and Central American *Cryptotermes* species, namely *C. mangoldi* Scheffrahn & Křeček, 1999, *C. parvifrons* Scheffrahn & Křeček, 1999, *C. cymatofrons* Scheffrahn & Křeček, 1999, *C. rotundiceps* Scheffrahn & Křeček, 1999, and *C. cavifrons* Banks, 1906 (Fig. 2).

### ﻿Taxonomy

#### ﻿ Cryptotermes mobydicki

Taxon classificationAnimaliaBlattodeaKalotermitidae

Scheffrahn sp. nov.

C973AD1A-CC16-5C98-B16C-F1840E396187

https://zoobank.org/E555EA6F-B1F3-4410-BBD3-8E3F8774890E

##### Diagnosis.

**Figure 1.**
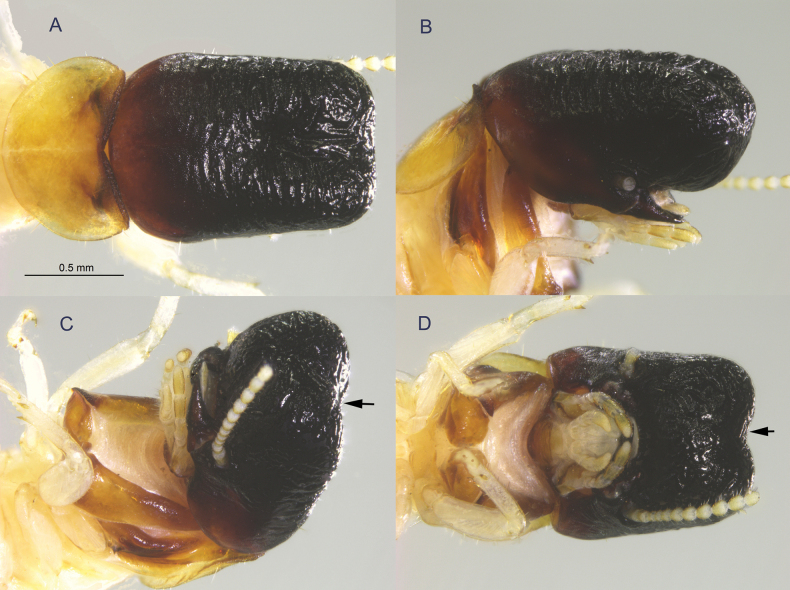
Holotype soldier of *Cryptotermes mobydicki* sp. nov. (FG1240), views of head and pronotum. A. Dorsal view; B. Lateral view; C. Oblique view; D. Ventral view. Arrows point to shallow incision of frontal process.

Among *Cryptotermes* soldiers worldwide, *C. mobydicki* is unique for the following characters: the head capsule is very long and narrow; the frontal flange and the frontal horns are absent; and, in dorsal view, the mandibles are greatly eclipsed by the extended frontal process (Fig. 1).

**Figure 2.**
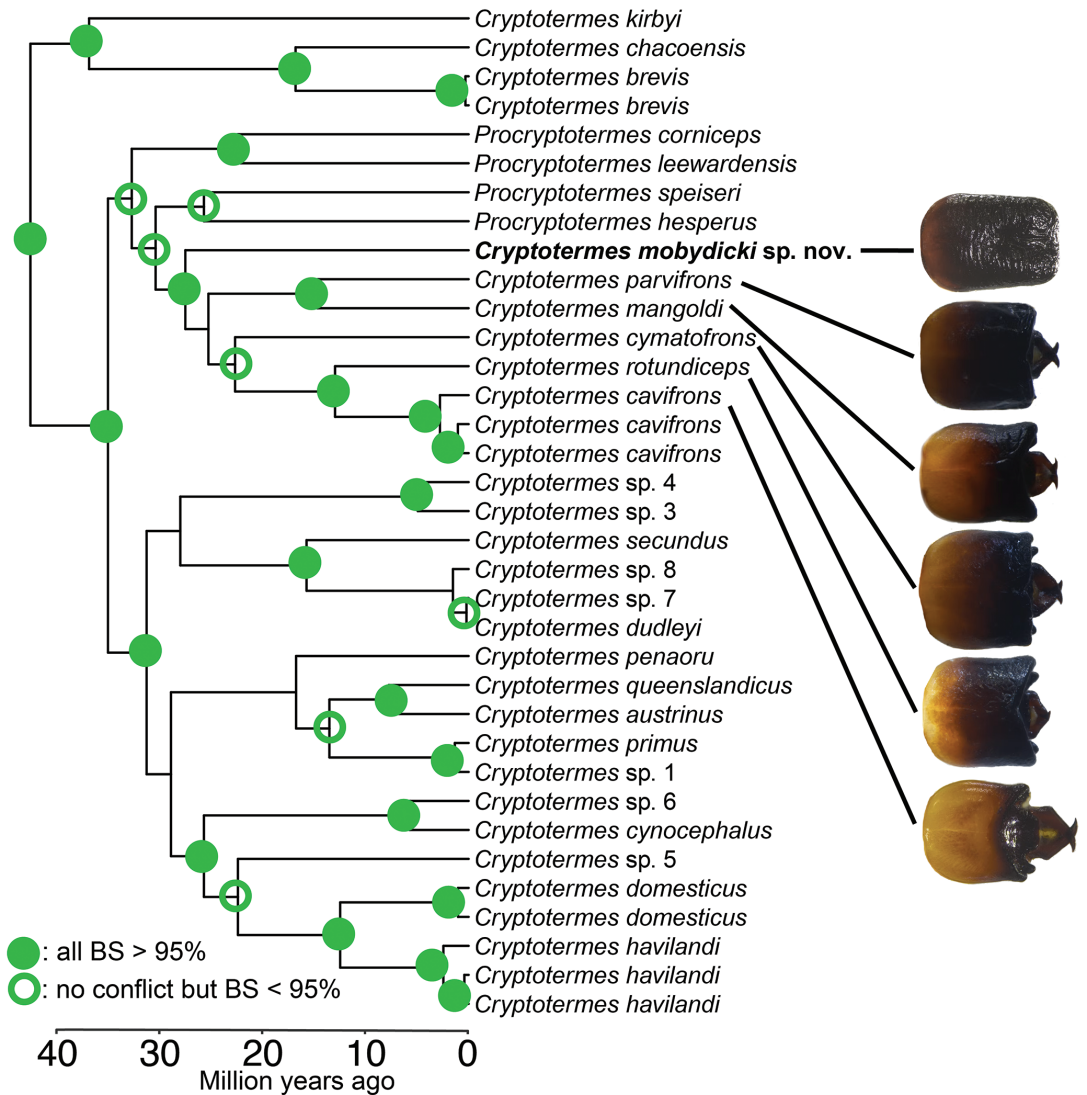
Bayesian summary support molecular phylogenetic tree showing relationship of *Cryptotermes mobydicki* sp. nov. and other *Cryptotermes* and *Procryptotermes* species. The branches with strong phylogenetic support, i.e. branches with more than 95% bootstrap support in all phylogenetic analysis and branches with bootstrap support lower than 95% but without conflict among all the summarized phylogenetic trees, are labeled with a full circle and empty circle, respectively. Photographs show dorsal view comparison of *C. mobydicki* sp. nov. and its most closely related *Cryptotermes* species from among the sampled species. The photographs are not to scale. The phylogenetic tree was extracted from the Kalotermitidae phylogeny reconstructed by Buček et al. (2022).

In the Bacchus (1987) key to *Cryptotermes* soldiers worldwide (at the time), the second couplet is amended as follows:

**Table:** 

2	Head index ratio LHCr1/HLW (length of head to cephalic ridge/maximum head width) = ca. 1.40	***C. mobydicki* sp. nov.**
–	Head index ratio 0.95–1.25	**3**
3	Head index ratio larger; LHCrl/HW = 1.18–1.25	***C. darwini***

In the key to South American *Cryptotermes* soldiers (Scheffrahn and Vasconcellos 2023), the first couplet is amended as follows:

**Table:** 

1	In dorsal view, anterior margin of head capsule consists of a frontal process only, without mandibles or genal horns visible, frontal horns absent; head capsule narrow and elongate	***C. mobydicki* sp. nov.**
–	In dorsal view, anterior margin of head capsule not obscuring mandibles or genal horns, frontal horns present; head capsule nearly quadrate	**2**
2	In dorsal (or ventral) view, genal horns form anterolateral knobs of head capsule; vertex smooth (introduced species) (fig. 3A, B)	**3**

##### Description.

***Imago*** unknown. ***Soldier*** (Fig. 1A–D). Head capsule, in dorsal view, black; elongate, narrowing to a blunt frontal process; a few setae along lateral margins. Frontal process and vertex embellished with wavy longitudinal rugosity. Occiput chestnut-brown, without rugosity. Pronotum yellowish orange; anterior margin darker, curving upward, weakly serrated; posterior and lateral margins evenly rounded with a few setae of variable lengths (Fig. 1A). In lateral view, frontal process curving slightly downward; eclipsing the mandible by about 1.4× the length of entire head capsule. Frontal flange (cephalic ridge) absent. Genal horns with weak curvature below antennal sockets; frontal horns absent (Fig. 1B). In oblique view, mandibles horn-like, curving inwards. Frontal process with shallow incision (Fig. 1C), best seen in ventral aspect (Fig. 1D). Antennae with 11 articles; article formula 2 > 3 = 4 < 5. Measurements in Table 1.

**Table 1.** Measurements (mm) of the two available soldier specimens of *Cryptotermes mobydicki* sp. nov.

Measurement	Holotype	Paratype
Head length	1.36	1.33
Head width, maximum	0.94	0.99
Head width, minimum (frontal process)	0.81	0.79
Head height, maximum excluding postmentum	0.74	0.77
Pronotum, maximum length	0.68	0.72
Pronotum, maximum width	0.89	0.91
Left mandible length, tip to ventral condyle	0.44	0.42

##### Type locality.

French Guiana, Petit Saut, near Sinnamary River (5.0684, −53.0429); 42 m a.s.l.; 11 Mar. 2016; D. Sillam-Dussès and J. Šobotník leg.

##### Type material.

***Holotype***: soldier, University of Florida Termite Collection (UFTC) no. FG1240 (in a separate vial with the remaining sample) (Fig. 1). ***Paratypes***: from the type locality, same data; 1 soldier and 10 pseudergates; UFTC no. FG1240.

##### Etymology.

Named after Moby Dick from Herman Melville’s classic novel. The lateral view of the soldier frontal process and elongate head (Fig. 1B) resembles the head of a sperm whale. Both organisms have mandibles eclipsed by the head, and the whale eye and soldier’s antennal socket are comparatively positioned.

##### Ecological note.

The small sample of *Cryptotermes mobydicki* sp. nov. colony was found in a dead standing tree (about 20 cm in diameter at chest height), near to its top, about 8 m above ground. Although it was recognized as potentially valuable sample, it was not possible to extract more material due to the wood hardness. Without the proper extraction tool (sharp hatchet per Scheffrahn et al. 2018), this sample would not have been accessible. The type locality is classified as tropical rainforest (Köppen-Geiger) with an annual precipitation of 275–300 cm.

## ﻿Discussion

*Cryptotermes mobydicki* sp. nov. is so unique among kalotermitids that RHS first thought it was in a new or different genus. Results of the phylogenetic reconstruction (Fig. 2) bore out its identity as a *Cryptotermes* species. It was also unexpected that *C. mobydicki* from French Guiana shares a clade with *C. mangoldi* from coastal Colombia and the Dominican Republic and *C. parvifrons* from Trinidad and Tobago and from the island of Grenada. The taxonomic sampling in Fig. 2 includes *C. mangoldi*, *C. brevis*, and *C. chacoensis* from among the 13 previously described endemic South American *Cryptotermes* species. The sister *Cryptotermes* species of *C. mobydicki* is likely going to be identified more precisely in future phylogenetic iterations with increased taxonomic sampling. Nevertheless, soldiers of other South American and Central American *Cryptotermes* soldiers having quadrate head capsules and projecting mandibles (Fig. 2), have no character affinity to *C. mobydicki* sp. nov., thus substantiating that soldier characters are highly derived (Kaji et al. 2016).

## Supplementary Material

XML Treatment for Cryptotermes mobydicki

## ﻿Additional information

### Use of AI

No use of AI was reported.

### Author ORCIDs

Rudolf H. Scheffrahn https://orcid.org/0000-0002-6191-5963

Aleš Buček https://orcid.org/0000-0001-9479-3141

David Sillam-Dussès https://orcid.org/0000-0001-5027-8703

Jan Šobotník https://orcid.org/0000-0002-8581-637X

### Data availability

All of the data that support the findings of this study are available in the main text.

## References

[B1] Bacchus S (1987) A taxonomic and biometric study of the genus *Cryptotermes* (Isoptera: Kalotermitidae). Tropical Pest Bulletin 7: 1–91.

[B2] Buček A Wang M Šobotník J Hellemans S Sillam-Dussès D Mizumoto N Stiblík P Clitheroe C Lu T González Plaza JJ Mohagan A Rafanomezantsoa J-J Fisher B Engel MS Roisin Y Evans TA Scheffrahn R Bourguignon T (2022) Molecular phylogeny reveals the past transoceanic voyages of drywood termites (Isoptera, Kalotermitidae). Molecular Biology and Evolution 39(5): msac093. 10.1093/molbev/msac093 PMC9113494 35511685

[B3] Casalla R Scheffrahn R Korb J (2016) *Cryptotermes colombianus* a new drywood termite and distribution record of *Cryptotermes* in Colombia. ZooKeys 596: 39–52. 10.3897/zookeys.596.9080 PMC4926653 27408575

[B4] Constantino R (2000b) A new *Cryptotermes* from the Brazilian Atlantic Forest (Isoptera: Kalotermitidae). Sociobiology 36: 525–530.

[B5] Constantino R (2002) The pest termites of South America: Taxonomy, distribution and status. Journal of Applied Entomology 126(7-8): 355–365. 10.1046/j.1439-0418.2002.00670.x

[B6] Constantino R (2020a) Termite Database. Brasília, University of Brasília. http://termitologia.net [Accessed 15 July 2025]

[B7] Emerson AE (1925) The termites of Kartabo, Bartica District, British Guiana. Zoologica (New York) 6(4): 291–459. 10.5962/p.190324

[B8] Kaji T Keiler J Bourguignon T Miura T (2016) Functional transformation series and the evolutionary origin of novel forms: Evidence from a remarkable termite defensive organ. Evolution & Development 18(2): 78–88. 10.1111/ede.12179 26766508

[B9] Krishna K Grimaldi DA Krishna V Engel MS (2013) Treatise on the Isoptera of the world. Vol. 2, basal families. Bulletin of the American Museum of Natural History 377(7): 201–623. 10.1206/377.2

[B10] Lee S-B Lee H Song J Jang BJ Cho SM Yum J Ahn N-H Kim J Lee H Choi Y-S Lee HM Seo MS Lee H Son S Bergbower H Lim K Su N-Y Lee W (2024) A post in an internet forum led to a discovery of an invasive drywood termite in Korea, *Cryptotermes domesticus* (Haviland) (Blattodea: Kalotermitidae). Journal of Integrated Pest Management 15(1): 34. 10.1093/jipm/pmae026

[B11] Light SF (1935) The Templeton Crocker Expedition of the California Academy of Sciences, 1932. No. 20. The termites. Proceedings of the California Academy of Sciences 21: 233–256.

[B12] Roisin Y (2003) *Cryptotermes chacoensis*, a new species from native South American inland habitats (Isoptera: Kalotermitidae). Sociobiology 42: 319–327.

[B13] Scheffrahn RH (1993) *Cryptotermes chasei*, a new drywood termite (Isoptera: Kalotermitidae) from the Dominican Republic. The Florida Entomologist 76(3): 500–507. 10.2307/3495649

[B14] Scheffrahn RH (2018) A new *Cryptotermes* (BlattodeaIsoptera): Kalotermitidae) from Honduras and known distribution of New World *Cryptotermes* species. The Florida Entomologist 101(4): 657–662. 10.1653/024.101.0403

[B15] Scheffrahn RH (2019) UF Termite database. University of Florida Termite Collection. https://www.termitediversity.org/ [Accessed 1 July 2025]

[B16] Scheffrahn RH (2021) *Cryptotermes camelus* (Isoptera: Kalotermitidae), a new drywood termite species from the Bolivian Chaco. Zootaxa 4938(1): 145–147. 10.11646/zootaxa.4938.1.9 33756990

[B17] Scheffrahn RH Křeček J Chase JA Su N-Y Krecek J (1998) *Cryptotermes abruptus*, a new drywood termite (Isoptera: Kalotermitidae) from southeastern Mexico. The Florida Entomologist 81(2): 188–193. 10.2307/3496085

[B18] Scheffrahn RH Křeček J Chase J Maharajh B Mangold J (2006) Taxonomy, biogeography, and notes on termites (Isoptera: Kalotermitidae, Rhinotermitidae, Termitidae) of the Bahamas and Turks and Caicos Islands. Annals of the Entomological Society of America 99(3): 463–486. 10.1603/0013-8746(2006)99[463:TBANOT]2.0.CO;2

[B19] Scheffrahn RH Chase JA Mangold JR Hochmair HH (2018) Relative occurrence of the family Kalotermitidae (Isoptera) under different termite sampling methods. Sociobiology 65(1): 88–100. 10.13102/sociobiology.v65i1.2097

[B20] Walker F (1853) List of the specimens of neuropterous insects in the collection of the British Museum. Part III (Termitidae–Ephemeridae). Trustees of the British Museum, London, 477–585.

